# Robot-assisted spinal surgery: technological maturity, clinical evidence, and future directions

**DOI:** 10.3389/frobt.2026.1883508

**Published:** 2026-09-10

**Authors:** Xue Li, Shilong Li, Minfei Wu, Jianhang Jiao, Tong Yu

**Affiliations:** 1 Department of Orthopaedic Medical Center, The Second Norman Bethune Hospital of Jilin University, Changchun, Jilin, China; 2 Department of Orthopedic and Soft Tissue Surgery, Jilin Cancer Hospital, Changchun, Jilin, China

**Keywords:** clinical evidence, pedicle screw placement, robot-assisted spinal surgery, spinal navigation, surgical robotics

## Abstract

Robot-assisted spinal surgery has evolved from bone-mounted trajectory guidance to integrated navigation and, in selected research systems, constrained task execution. This evolution has been accompanied by rapid commercial and academic activity, but regulatory status, technical capability, and clinical effectiveness are often conflated. Such conflation can overstate both the maturity of emerging platforms and the certainty of their patient-level benefits. This structured narrative review was developed from a reproducible PubMed search, targeted citation chasing, Crossref metadata verification, and model-specific regulatory checks. Evidence was organized by source class and by outcome domain rather than by manufacturer chronology alone. Established platforms were separated from early clinical and preclinical systems, and regulatory clearance or registration was treated as distinct from evidence of comparative effectiveness. The most consistent evidence concerns pedicle-screw placement accuracy. Comparative studies and evidence syntheses generally favor robot-assisted placement over freehand or fluoroscopy-guided techniques, although the magnitude and consistency of benefit vary by platform, comparator, grading system, and surgeon experience. Radiation exposure may be reduced in some workflows, but acquisition protocols and reporting units are heterogeneous. Evidence for operative time, complications, revision surgery, patient-reported outcomes, and cost-effectiveness remains inconsistent or sparse. The principal contribution of this review is an evidence-maturity framework that links each technical capability to the strongest available clinical evidence and explicitly separates premarket regulatory decisions from claims of clinical superiority. Robot-assisted systems should currently be understood as surgeon-controlled enabling technologies. Future progress will depend less on adding degrees of freedom than on demonstrating reproducible patient benefit, safe task autonomy, interoperable data pipelines, and value across health-care settings.

## 1 Introduction

Spinal instrumentation requires accurate three-dimensional localization because pedicles and other osseous targets lie close to neural and vascular structures. Freehand and fluoroscopy-guided techniques remain effective in experienced hands, but their performance can be influenced by anatomy, imaging quality, surgeon experience, and cumulative radiation exposure. Navigation and robotic assistance were developed to improve the reproducibility of planned trajectories and to support minimally invasive workflows (Overley et al., 2017; Łajczak et al., 2024; Vo et al., 2020).

The term “spinal robot” covers devices with substantially different functions. Some systems position a guide according to a preoperative computed-tomography plan. Others integrate optical tracking, intraoperative imaging, navigated instruments, and a rigid robotic arm. A smaller group of research systems performs constrained components of an operation, such as K-wire insertion or laminectomy, under surgeon supervision. These categories should not be treated as equivalent because their failure modes, regulatory indications, and evidentiary requirements differ.

Clinical adoption also remains uneven. An international AO Spine survey reported that robotic assistance was used less frequently than conventional navigation and identified acquisition cost and limited perceived benefit as major barriers (Motov et al., 2025). At the same time, evidence syntheses report mixed, platform-dependent technical findings: some analyses favor robot-assisted accuracy, whereas others do not, and confidence is often limited by heterogeneity and the quality of the underlying reviews (Sun et al., 2024; Li et al., 2025; Wei et al., 2022; Fu et al., 2021). This gap between technical performance and demonstrated patient benefit is central to interpreting the field.

Several prior reviews have described individual systems or summarized screw-placement studies (Overley et al., 2017; Łajczak et al., 2024; Vo et al., 2020). The present review uses a different organizing principle. It separates three questions that are frequently merged: (i) what a platform is technically designed to do, (ii) what a regulator has cleared or registered for a specified model and indication, and (iii) what comparative clinical evidence shows. It then grades the maturity of evidence across radiographic, perioperative, patient-centered, and economic outcomes. This evidence-to-capability mapping is intended to reduce unsupported extrapolation from device availability or technical feasibility to clinical superiority.

## 2 Review design and literature selection

### 2.1 Review type and scope

This article is a structured narrative review rather than a systematic review or meta-analysis. Its scope includes robot-assisted technologies used for spinal instrumentation, navigation, and selected bone-work tasks. The review covers technical evolution, model-specific regulatory records, clinical outcomes, implementation barriers, and future development. It does not claim exhaustive identification of every marketed device, does not estimate a pooled treatment effect, and does not provide a formal risk-of-bias or GRADE assessment.

### 2.2 Search strategy and source verification

PubMed was searched on 1 August 2026 for publications dated from 1 January 2000 through 30 June 2026. The final discovery query was: (“robot-assisted spine surgery” [Title/Abstract] OR “robotic spine surgery” [Title/Abstract] OR (robot*[Title/Abstract] AND “pedicle screw” [Title/Abstract])). The date restriction was applied using the PubMed publication-date field. This query returned 792 records at the time of the search. Backward citation chasing from relevant reviews and forward identification of newer platform-specific studies were used to locate additional sources.

Crossref was used for DOI-exact first-pass verification of author order, article title, journal, year, volume, issue, pagination or article number, and digital object identifier. DOI-exact PubMed or PubMed Central records and publisher pages were then used for independent confirmation; when deposits disagreed, the published article record and the discrepancy were documented. Regulatory statements were checked against model-specific records in the US Food and Drug Administration 510(k) database. Chinese regulatory status was reported only when a model- and indication-specific record could be distinguished; a single approval date was not generalized across the TiRobot product family or across indications. Manufacturer webpages were not used as evidence of clinical effectiveness.

### 2.3 Selection rules

Sources were selected purposively for predefined domains: platform architecture; regulatory status; screw-placement accuracy; radiation exposure; operative workflow; complications and revision; patient-reported outcomes; economic evidence; learning curves; and emerging autonomy. Eligible clinical sources included evidence syntheses, randomized or prospective comparative studies, retrospective comparative studies, and informative platform-specific case series. Cadaveric, preclinical, or conference-abstract evidence was included only to describe emerging capabilities and was labeled accordingly.

Sources were excluded when they concerned a non-spinal specialty, described a different platform with a similar acronym, provided only generic robotics background for a platform-specific claim, or supported a commercial or historical statement that was not necessary to the scientific synthesis. This rule led to removal of the urological review by Brassetti et al., the MIDLIF-versus-TLIF study by Silva et al., and the general “Robot System Assistant” paper by Strazdas et al. from the revised reference set because none supports the spinal-platform claims for which it had been cited.

The final synthesis retained 48 peer-reviewed publications and five model-specific FDA records. Because the review is narrative, the 792-record discovery set was not processed through a PRISMA flow diagram, duplicate independent screening was not performed, and reasons for exclusion were not enumerated for every record. These limitations are stated explicitly to prevent the search description from being interpreted as a systematic-review protocol.

### 2.4 Evidence hierarchy and synthesis method

**FIGURE 1:**
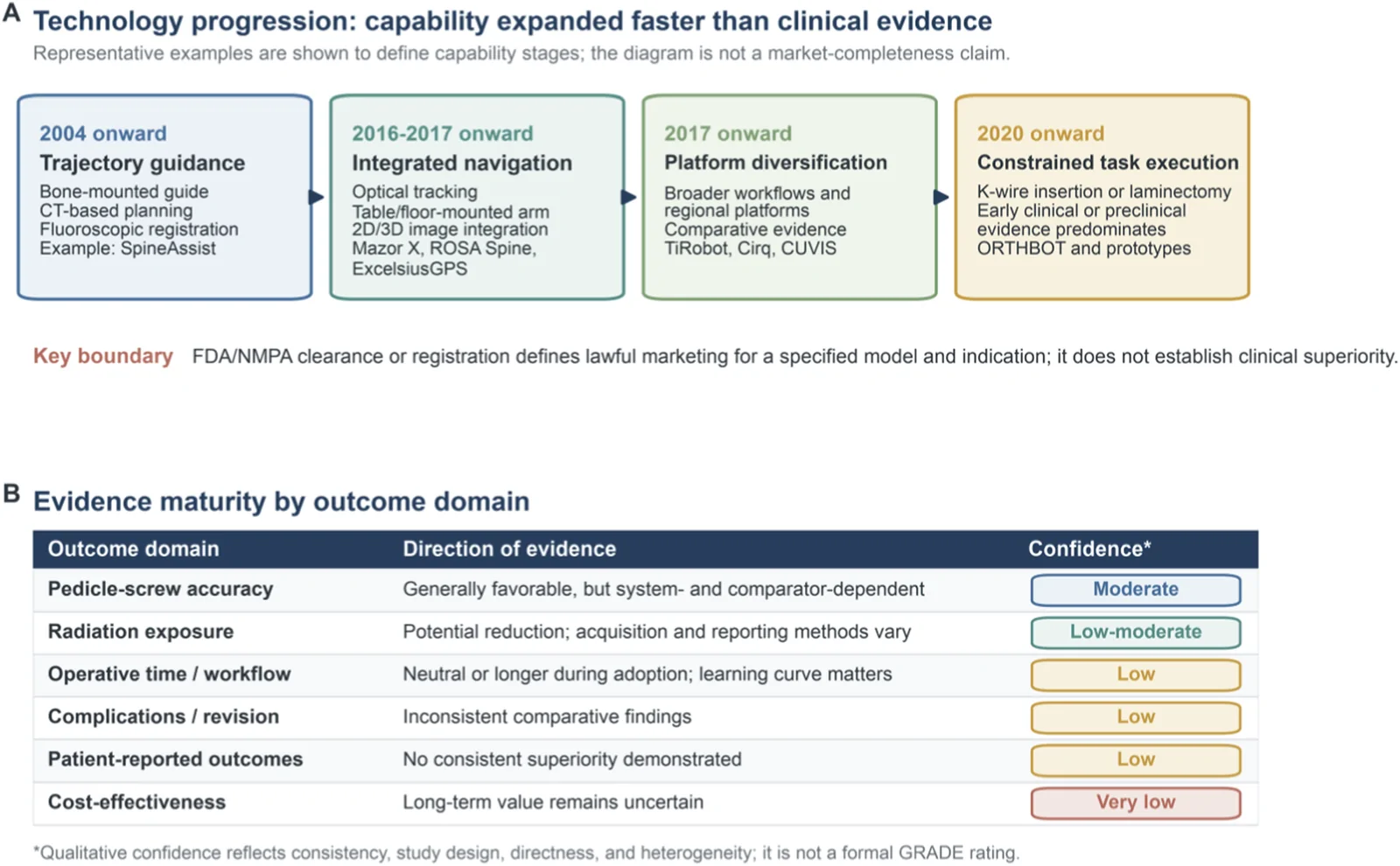
**(A,B)** Maps technical progression against outcome-specific evidence maturity. The figure was drawn *de novo* in Python from the evidence summarized in this review and the model-specific regulatory records (U.S. Food and Drug Administration, 2004; U.S. Food and Drug Administration, 2016; U.S. Food and Drug Administration, 2017a; U.S. Food and Drug Administration, 2017b; U.S. Food and Drug Administration, 2021). No third-party photographs, logos, or copyrighted product renderings were used; therefore, no external image permission is required. Premarket regulatory decisions are model- and indication-specific and do not establish comparative clinical superiority. The qualitative confidence labels are not formal GRADE ratings.

Evidence was assigned to five source classes: class A, umbrella reviews, systematic reviews, or meta-analyses; class B, randomized or prospective comparative studies; class C, retrospective comparative studies; class D, single-arm clinical series or technical reports; and class E, cadaveric, preclinical, conference-abstract, or regulatory sources. Regulatory records were used only to verify device identity, indication, and decision date, not to infer effectiveness. Figure 1 maps technical progression against outcome-specific evidence maturity.

For each outcome domain, confidence was judged qualitatively from consistency, study design, directness, platform specificity, and heterogeneity. The labels “moderate,” “low-moderate,” “low,” and “very low” are narrative descriptors and are not formal GRADE ratings. Table 1 separates representative platforms by architecture and source class, and Table 2 presents the outcome-domain synthesis.

**TABLE 1:** Representative robot-assisted spinal systems, model-specific regulatory sources, and evidence maturity.

Platform	Architecture and imaging	Selected regulatory record	Evidence class	Bounded interpretation	Sources
SpineAssist	Bone-mounted miniature guide; CT planning; fluoroscopic registration	FDA K033413; decision 7 January 2004	D; later comparative evidence	Early trajectory-guidance platform; surgeon performs the operative task	(Lieberman et al., 2006; U.S. Food and Drug Administration, 2004)
Mazor X	Table-mounted arm; robotic trajectory guidance with navigation	FDA K163221; decision 4 April 2017	C-D	Integrated planning and navigation; evidence is configuration- and workflow-specific	(Lee et al., 2023; O’Connor et al., 2021; U.S. Food and Drug Administration, 2017b)
ROSA spine	Computer-controlled arm; 3D planning; stable instrument-holder orientation	FDA K151511; decision 4 January 2016	D	FDA indication is model- and approach-specific; not interchangeable with other ROSA products	(Lefranc and Peltier, 2016; U.S. Food and Drug Administration, 2016)
ExcelsiusGPS	Floor-mounted rigid arm; integrated navigation; compatible 2D/3D imaging	FDA K171651; decision 16 August 2017	C-D	Technical and early clinical reports support screw placement; evidence is protocol- and institution-specific	(Zygourakis et al., 2018; Kanaly et al., 2023; U.S. Food and Drug Administration, 2017a)
TiRobot/TiRobot II	Floor-mounted 6-degree-of-freedom arm; optical tracking	China; registration is model- and indication-specific	B-C	Spine evidence includes prospective and comparative studies; no single approval date is generalized to the family	(Han et al., 2019; Tian et al., 2017; Yan et al., 2022)
Cirq	Compact alignment arm integrated with navigation	FDA K210989; decision 12 November 2021	C-D	Spine-specific case series and comparative evidence; 2021 record corrects the prior 2019 claim	(Gabrovsky et al., 2023; Chesney et al., 2022; Desai and Adams, 2022; U.S. Food and Drug Administration, 2021)
CUVIS-spine	Floor-mounted arm with optical tracking and planning	Jurisdiction-specific; not generalized here	D	Multicenter clinical evaluation exists, but regulatory and commercial claims require model-specific sources	(Ha et al., 2023)
ORTHBOT	Independent arm; supervised automatic K-wire guidance and placement	Jurisdiction-specific; not generalized here	B-D	Clinical studies concern constrained K-wire tasks, not unrestricted autonomous surgery	(Li et al., 2020; Li et al., 2021a; Li et al., 2021b)
Laminectomy prototype	Robotic arm, force sensing, ultrasonic osteotome, optical tracking	Preclinical research system	E	Cadaveric feasibility for constrained bone removal; not evidence of clinical effectiveness	(Li et al., 2021; Li et al., 2023)

FDA, dates refer to substantial-equivalence decisions, not premarket approval. Regulatory decisions are jurisdiction-, model-, and indication-specific; they do not establish comparative effectiveness or current commercial availability. Evidence class: A, evidence synthesis; B, randomized or prospective comparative study; C, retrospective comparative study; D, single-arm clinical or technical report; E, cadaveric, preclinical, conference-abstract, or regulatory source.

**TABLE 2:** Outcome-domain evidence synthesis. Confidence labels are qualitative narrative judgments based on consistency, study design, directness, platform specificity, and heterogeneity; they are not formal GRADE ratings.

Outcome	Highest source class	Direction of evidence	Confidence	Main limitations	Sources
Pedicle-screw accuracy	A-B	Generally favorable; platform- and comparator-dependent	Moderate	Heterogeneous grading scales, comparators, anatomy, and study quality	(Sun et al., 2024; Wei et al., 2022; Fu et al., 2021; Han et al., 2019; Kim et al., 2017; Laudato et al., 2018; Solomiichuk et al., 2017)
Radiation exposure	A-C	Potential reduction in selected workflows	Low-moderate	Patient and staff exposure often conflated; inconsistent units and imaging protocols	(Wei et al., 2022; Fu et al., 2021; Wang et al., 2023; Solomiichuk et al., 2017)
Operative time/workflow	A-C	Neutral or longer during adoption; may improve with experience	Low	Different definitions of setup, robot, fluoroscopy, and total operating time	(Lee et al., 2023; Wang et al., 2023; Pennington et al., 2022)
Complications/revision	C	Inconsistent comparative findings	Low	Confounding, coding differences, case complexity, and follow-up duration	(Liounakos et al., 2021; Malik et al., 2021; De Biase et al., 2021; Yang et al., 2020)
Patient-reported outcomes	A-B	No consistent robot-attributable advantage	Low	Technical accuracy may have a ceiling effect; studies not powered for patient outcomes	(Fu et al., 2021; Wang et al., 2023)
Cost-effectiveness	C-D	Index cost often higher; long-term value uncertain	Very low	Capital amortization, volume, maintenance, revision, and quality of life incompletely measured	(Motov et al., 2025; Ezeokoli et al., 2022)

## 3 Technological evolution and representative platforms

### 3.1 From bone-mounted guidance to integrated navigation

SpineAssist established an early architecture for miniature bone-mounted robotic guidance. The system translated a computed-tomography-based preoperative plan into the orientation of a mechanical guide fixed relative to the patient. The original FDA record, K033413, received a substantial-equivalence decision on 7 January 2004, and the technical development was subsequently described in peer-reviewed reports (Lieberman et al., 2006; U.S. Food and Drug Administration, 2004). The scientific importance of this platform lies in demonstrating that a planned trajectory could be transferred through a compact mechanical guide; it does not by itself establish superiority over other placement methods.

Later Mazor platforms shifted toward larger table-mounted architectures, greater imaging integration, and real-time navigation. The FDA record for Mazor X, K163221, received a substantial-equivalence decision on 4 April 2017 (U.S. Food and Drug Administration, 2017b). Comparative and technical studies have examined differences between Renaissance and Mazor X and have described the integration of robotic trajectory guidance with navigation (Lee et al., 2023; O’Connor et al., 2021). These reports support statements about workflow and system architecture, but clinical outcomes must still be interpreted from comparative studies rather than from product generation alone.

ROSA Spine uses a computer-controlled electromechanical arm to orient an instrument holder according to three-dimensional planning. FDA record K151511 received a substantial-equivalence decision on 4 January 2016 and specifies lumbar pedicle-screw placement through a posterior approach (Lefranc and Peltier, 2016; U.S. Food and Drug Administration, 2016). This indication-specific wording is important: the existence of related ROSA products in neurosurgery or arthroplasty should not be used to broaden claims about the spine platform.

ExcelsiusGPS integrates navigation with a rigid floor-mounted robotic arm and supports compatible imaging and instrument workflows. FDA record K171651 received a substantial-equivalence decision on 16 August 2017 (U.S. Food and Drug Administration, 2017a). Published technical and clinical series describe pedicle-screw placement during early clinical adoption (Zygourakis et al., 2018; Kanaly et al., 2023). As with other platforms, evidence from one imaging protocol or institution should not be generalized to all configurations.

### 3.2 Platform diversification

TiRobot and TiRobot II are optical-tracking orthopedic robotic platforms that have been evaluated in spinal instrumentation studies, including prospective and comparative work (Han et al., 2019; Tian et al., 2017; Yan et al., 2022). The product family has also been used for non-spinal orthopedic indications. Because registration is model- and indication-specific, the revised review does not assign a single NMPA approval year to every TiRobot configuration. This narrower wording replaces the previous unsupported generalization.

Cirq is a compact alignment arm integrated with Brainlab navigation. The model family and its cranial-and-spine indication appear in FDA record K210989, which received a substantial-equivalence decision on 12 November 2021 (U.S. Food and Drug Administration, 2021). This corrects the earlier statement that Cirq was FDA cleared in 2019. Spine-specific reports include feasibility, accuracy, and comparative series (Gabrovsky et al., 2023; Chesney et al., 2022; Desai and Adams, 2022).

CUVIS-spine and ORTHBOT illustrate further diversification. A multicenter study evaluated a new robotic system associated with CUVIS-spine, whereas randomized and multicenter studies of an independent robotic arm evaluated guided or automated K-wire placement (Ha et al., 2023; Li et al., 2020; Li et al., 2021a; Li et al., 2021b). These studies provide platform-specific clinical evidence, but their results do not justify claims of unrestricted autonomous surgery. Table 1 therefore describes their evidence maturity without inferring a universal regulatory status across jurisdictions.

### 3.3 Constrained task execution and emerging autonomy

Most clinically established systems position or constrain an instrument guide while the surgeon performs drilling, tapping, or screw insertion. A smaller set of systems has moved toward constrained task execution. ORTHBOT studies evaluated robotic K-wire placement, while a separate research platform combined a robotic arm, force sensing, and an ultrasonic osteotome for autonomous laminectomy in cadaveric models (Li et al., 2020; Li et al., 2021a; Li et al., 2021b; Li et al., 2021; Li et al., 2023). These tasks remain narrow, structured, and supervised.

Reviews of full robotic automation emphasize persistent limitations in tracking and registration and the continuing need for human decision-making (Samprón et al., 2025). A conference abstract describing a supervisory controlled seven-degree-of-freedom robot with optical coherence tomography guidance for porcine pedicle instrumentation is appropriately treated as early evidence rather than proof of an established commercial platform (Johnston et al., 2024). Accordingly, the revised manuscript removes unsupported claims about 8i Robotics, ALAYA, and Stealth AXiS when no claim-matched peer-reviewed or model-specific regulatory source was available in the audited set.

## 4 Clinical evidence by outcome domain

### 4.1 Pedicle-screw accuracy

Pedicle-screw accuracy is the most extensively studied outcome. Network and conventional meta-analyses generally report a higher probability of ideal or clinically acceptable placement with robotic assistance than with freehand or fluoroscopy-guided techniques, but results differ by platform and grading definition (Wei et al., 2022; Fu et al., 2021). Randomized and prospective studies of TiRobot and other platforms also report high placement accuracy (Han et al., 2019; Kim et al., 2017; Fan et al., 2022).

The interpretation is nevertheless less definitive than a simple “robot versus freehand” comparison suggests. An overview of systematic reviews found that the underlying reviews were of low or critically low methodological quality and concluded that current evidence was insufficient for strong clinical-guideline recommendations (Sun et al., 2024). A later umbrella review reported potential benefits but predominantly low-to-very-low certainty and substantial heterogeneity (Li et al., 2025). The strongest defensible conclusion is therefore that robotic assistance generally improves or preserves radiographic accuracy in many settings, with moderate confidence, rather than that every platform is superior in every procedure.

Platform-specific comparative studies reinforce this qualification. Robotic guidance has been compared with O-arm navigation, fluoroscopy, and freehand placement with different conclusions (Laudato et al., 2018; Wang et al., 2023; Solomiichuk et al., 2017; Du et al., 2020; Molliqaj et al., 2017). Differences in screw-grading scales, spinal region, deformity severity, image acquisition, and surgeon experience limit cross-study comparison. Accuracy should also be separated from clinical importance: a radiographic improvement does not automatically translate into fewer neurological complications, better function, or fewer revisions.

### 4.2 Radiation exposure

Radiation exposure is commonly presented as a robotic advantage, but the direction and magnitude of effect depend on what is measured. Studies variously report fluoroscopy time, dose-area product, patient dose, surgeon dose, or imaging events. Preoperative computed tomography, intraoperative three-dimensional imaging, and fluoroscopic registration can redistribute exposure rather than uniformly reduce it. Evidence syntheses suggest a potential reduction in selected robot-assisted workflows, but heterogeneity is substantial (Wei et al., 2022; Fu et al., 2021).

The revised interpretation therefore avoids a universal claim. Robot-assisted workflows may reduce occupational fluoroscopy exposure when planning and three-dimensional imaging replace repeated two-dimensional shots, but patient dose can vary according to the imaging protocol. Future studies should report patient and operating-room personnel exposure separately and use standardized units.

### 4.3 Operative time, workflow, and learning curves

Robotic systems add planning, registration, calibration, draping, and verification steps. These steps can prolong early cases or lead to abandonment when registration or positioning fails. With experience, some components become faster, but learning is platform- and workflow-specific. A systematic review of learning curves found heterogeneity in definitions and proposed more consistent reporting (Pennington et al., 2022). Comparisons involving different systems and navigation workflows have also produced mixed estimates of operative time (Lee et al., 2023; Wang et al., 2023). A robotic series found comparable placement accuracy between residents and an attending surgeon, but it was not a robot-versus-nonrobot comparison (Vardiman et al., 2020).

Operative time should therefore not be used as a stand-alone measure of efficiency. Set-up time, robot time per screw, fluoroscopy time, conversion or abandonment, staff workload, and room turnover should be reported separately. The relevant implementation question is whether the complete pathway becomes more reliable and resource-efficient after training, not merely whether one intraoperative interval becomes shorter.

### 4.4 Complications, revisions, and patient-reported outcomes

Perioperative complications in spine surgery are heterogeneous and can include dural injury, infection, neurological injury, hardware-related events, and medical complications (Farshad et al., 2020; Swann et al., 2016). Some robot-assisted cohorts have reported fewer complications or revisions, whereas other comparative studies found no difference or a higher coded complication burden (Liounakos et al., 2021; Malik et al., 2021; De Biase et al., 2021; Yang et al., 2020). These findings are difficult to reconcile because of confounding by indication, case complexity, coding practices, surgeon experience, and follow-up duration.

Patient-reported outcomes are even less consistently studied. A meta-analysis found no significant difference in patient-reported scores, and a 2-year comparative fusion study found no VAS or ODI advantage attributable to robotic assistance despite improvement in both groups (Fu et al., 2021; Wang et al., 2023). The most defensible conclusion is that evidence for complications, revision, and patient-reported outcomes remains low confidence. These outcomes should not be inferred from screw-placement accuracy.

### 4.5 Cost and adoption

Acquisition, service contracts, disposable instruments, imaging, training, and operating-room time all contribute to the cost of robot-assisted surgery. A matched cohort analysis found higher index costs for robot-assisted pedicle-screw placement (Ezeokoli et al., 2022), while the AO Spine survey identified acquisition cost as the dominant reported adoption barrier (Motov et al., 2025). Whether these expenditures translate into favorable long-term cost-effectiveness remains uncertain. Potential savings from shorter stays or fewer revisions are plausible but cannot be assumed without robust longitudinal evidence.

The AO Spine survey provides a complementary implementation perspective: cost was the dominant reported barrier and regular use remained limited (Motov et al., 2025). Economic evaluations should therefore include capital amortization, case volume, staff time, maintenance, consumables, reoperation, health-related quality of life, and opportunity costs. Until these components are measured prospectively, claims of cost-effectiveness remain very low confidence.

## 5 Limitations and requirements for clinical translation

Registration and tracking remain central technical vulnerabilities. Patient movement, reference-frame disturbance, instrument deflection, skiving, and line-of-sight obstruction can create a discrepancy between the plan and the operative anatomy. Robotic constraint may improve trajectory reproducibility, but it does not eliminate the need for surgeon verification. Robotic platforms remain shared-control systems, and surgeons must anticipate platform-specific technical pitfalls (Lee et al., 2025). Systems should provide transparent accuracy checks, clear abort criteria, and a rapid fallback workflow.

The evidence base has equally important limitations. Many studies are single-center, retrospective, and conducted during different phases of the learning curve. Comparators vary among freehand, fluoroscopy, and navigation. Outcomes are reported per screw, per level, or per patient, and the same cohort may contribute to multiple publications or evidence syntheses. These features can exaggerate precision and complicate pooling.

Platform-specific evidence also ages quickly because hardware, software, instruments, and regulatory indications change. A study of one software version should not be assumed to apply to later or earlier versions. Likewise, a model-specific FDA substantial-equivalence decision should not be described as approval of an entire company’s product family. Table 1 uses model-specific records and explicitly limits the inference drawn from them.

Finally, improved technical execution can have a ceiling effect. When conventional placement is already highly accurate, a small radiographic difference may not produce a detectable change in complications or function. Trials should therefore predefine clinically meaningful endpoints and recruit populations in whom a technical advantage could plausibly affect outcomes, such as complex deformity, minimally invasive multilevel instrumentation, or anatomically difficult trajectories.

## 6 Future directions

Artificial intelligence has been applied to preoperative three-dimensional modeling and planning, intraoperative image segmentation and navigation, and prediction of surgical outcomes or complications (Han et al., 2024; Zeng and Fu, 2024). These applications should be evaluated as decision-support components with defined inputs, failure modes, and calibration. Training data, independent validation, and version control should be reported before performance claims are generalized across institutions.

Progress toward semi-autonomous surgery will require more than a robotic arm. Safe task execution depends on reliable sensing, tracking and registration, task-state estimation, and supervisory control; current reviews emphasize persistent limitations and the continuing need for human decision-making (Samprón et al., 2025). The surgeon must retain situational awareness and the ability to pause or override the system. Early K-wire studies, cadaveric autonomous-laminectomy work, and an optical-coherence-tomography-guided porcine pedicle-instrumentation abstract demonstrate feasibility for narrow tasks, but evidence remains class D or E (Li et al., 2020; Li et al., 2021b; Li et al., 2021a; Li et al., 2021; Li et al., 2023; Johnston et al., 2024).

Augmented reality, optical coherence tomography, and other sensing modalities may supplement conventional optical tracking. Their value will depend on registration accuracy, latency, ergonomics, and integration with existing workflows. A new interface should not be treated as clinically superior until it improves a prespecified outcome without introducing new failure modes.

Surgical data science offers a broader path toward learning systems (Maier-Hein et al., 2017). Standardized logs of plans, registration errors, instrument trajectories, conversions, and outcomes could support post-market surveillance and continuous improvement. Such datasets also create governance obligations concerning privacy, interoperability, bias, cybersecurity, and accountability.

## 7 Discussion

The field has advanced substantially in mechanical stability, navigation integration, and workflow support. However, technical maturity and evidentiary maturity are not synchronized. The transition from a planned trajectory to a constrained clinical task is real, but most clinical evidence still concerns screw accuracy. Evidence becomes progressively weaker for radiation, time, complications, patient-reported outcomes, and cost-effectiveness.

This distinction explains why apparently conflicting conclusions can coexist. A platform can be technically accurate, lawfully marketed for a defined indication, and useful to an experienced team without having demonstrated superiority in long-term patient outcomes. Conversely, the absence of a proven patient-reported advantage does not mean that the technology has no value in selected complex cases. The appropriate conclusion is conditional rather than promotional.

The review’s evidence-maturity framework is intended to make these conditions explicit. It links platform claims to source class, separates premarket regulatory records from effectiveness evidence, and organizes clinical findings by outcome domain. This framework also clarifies what future studies must add: prospective multicenter designs, version-specific reporting, standardized radiation and workflow metrics, clinically meaningful endpoints, and full economic evaluation.

This review has limitations. The search was structured and reproducible but not systematic; the PubMed discovery set was not exhaustively screened, and no formal risk-of-bias assessment was performed. The rapidly changing device landscape makes any platform inventory time-sensitive. Regulatory records were verified for selected FDA-cleared models, but the review is not a global regulatory catalogue. Finally, the qualitative confidence labels reflect narrative judgment rather than a formal grading instrument.

## 8 Conclusion

Robot-assisted spinal surgery should currently be viewed as a family of surgeon-controlled enabling technologies with different levels of technical and evidentiary maturity. The most credible benefit is improved or preserved pedicle-screw placement accuracy in many settings. Potential reductions in radiation are workflow-dependent, while effects on operative time, complications, revision surgery, patient-reported outcomes, and cost-effectiveness remain uncertain.

Future progress should be judged by reproducible patient benefit and safe system behavior rather than by product generation or autonomy claims alone. Model-specific regulatory language, version-specific technical reporting, multicenter comparative studies, standardized outcomes, and transparent economic analyses are necessary to define where robotic assistance adds meaningful value to contemporary spinal surgery.
